# Supplementary material for: Current trends in the epidemiology of multidrug-resistant and beta-lactamase-producing Pseudomonas aeruginosa in Asia and Africa: a systematic review and meta-analysis
Source: PeerJ. 2025 Feb 24;13:e18986. doi: 10.7717/peerj.18986 (PMC11867037; doi:10.7717/peerj.18986)
Supplement: Supplemental Information 1 — ND, no data; KB, Kirby-Bauer disk diffusion; DDST, double disk synergy test; PCR, polymerase chain reaction; AMC, amoxicillin and clavulanic acid; AMK, amikacin; AMP, ampicillin; AMX, amoxicillin; AZT, aztreonam; CAZ, ceftazidime; CAR, carbenicillin; CEP, cephalothin; CFM, cefixime; CFR, cefadroxil; CFX, cefuroxime; CFZ, cefazolin; CHL, chloramphenicol; CIP, ciprofloxacin; COL, colistin/polymyxin E; CPD, cefpodoxime; CPM, cefepime; CTR, ceftriaxone; CTX, cefotaxime; CXT, ceftolozane and tazobactam; CZA, ceftazidime and tazobactam; DOR, doripenem; DOX, doxycycline; ETP, ertapenem; ERY, erythromycin; FOS, fosfomycin; FOX, cefoxitin; FUS, fusidic acid; GEN, gentamicin; IMI, imipenem; LEV, levofloxacin; MEM, meropenem; MIN, minocycline; NAL, nalidixic acid; NIT, nitrofurantoin; NOR, norfloxacin; OFX, ofloxacin; OXA, oxacillin; PIP, piperacillin; POL, polymyxin B; SAM, ampicillin and sulbactam; SXT, sulfamethoxazole and trimethoprim; TCC, ticarcillin and clavulanic acid; TET, tetracycline; TGC, tigecycline; TIC, ticarcillin; TMP, trimethoprim; TOB, tobramycin; TZP, piperacillin and tazobactam [file peerj-13-18986-s001.docx]

| **No** | **Study ID (author, year)** | **Study period** | **Country** | **Sample population** | **Age** | **Gender** | **Sample size** | **Study methods** | **Tested antibiotics** |
| --- | --- | --- | --- | --- | --- | --- | --- | --- | --- |
| 1 | Abavisani, M. 2021 | 2019 to 2020 | Iran | ND | All group | 37 M, 33 F | 70 | KB, Broth microdilution, PCR | AMK, AZT, CPM, CAZ, CIP, DOR, GEN, IMI, LEV, MEM, PIP, TOB, TZP |
| 2 | Abdelaziz, S. M. 2021 | 1/2018 to 2/2019 | Egypt | 194 | ND | ND | 58 | KB, Broth microdilution, DDST, PCR | AMK, GEN, TOB, AMX, AMC, CFR, CFX, CTX, CPM, MEM, AZT, CIP, LEV, DOX, TET, SXT |
| 3 | Abdeta, A. 2021 | 6/2019 to 5/2020 | Ethiopia | 429 | ND | 233 M, 196 F | 36 | KB | TZP, CAZ, CPM, IMI, MEM, CIP, TET, GEN, TOB, AMK |
| 4 | Ahmed, N. 2022 | 1/2018 to 7/2018 | Pakistan | 300 | All group | 189 M, 111 F | 32 | KB, DDST | AMP, AMK, CTR, CFX, CHL, CIP, GEN, IMI, MEM, TET, LEV, TOB, FUS, CFM, COL, POL |
| 5 | Alabdali, Y. A. J. 2021 | 7/2019 to 1/2020 | Iraq | 100 | All group | ND | 80 | KB, PCR | CAZ, AZT, TCC, TZP, AMC, AMK, ERY, CIP, MEM |
| 6 | Ali, F. 2021 | 1/2018 to 12/2018 | Pakistan | 1369 | All group | 57 M, 69 F | 126 | KB, DDST | CIP, PEF, LEV, NAL, NOR, FOX, CTX, CAZ, AMK, GEN, TOB, MEM, IMI, AZT, NIT, CHL, TGC, AMP, SXT, DOX, FOS, AMC, TZP |
| 7 | Ali, F. 2022 | 7/2019 to 12/2019 | Pakistan | 220 | All group | 21 M, 16 F | 37 | KB, DDST | CIP, LEV, CAR, CTR, CTX, CAZ, AMK, GEN, MEM, IMI, AZT, TZP |
| 8 | Alnimr, A. M. 2020 | 12/2018 to 4/2019 | Saudi Arabia | ND | All group | 43 M, 24 F | 67 | VITEK 2 | CAZ, CPM, TZP, CIP, LEV, GEN, AMK, IMI, MEM, CZA, CXT |
| 9 | Bitew, A. 2023 | 1/2019 to 7/2020 | Ethiopia | 156 | All group | 68 M, 88 F | 7 | VITEK 2 | AMP, AMC, TZP, CEP, CFZ, CFX, FOX, CPD, CAZ, CTR, CPM, GEN, TOB, CIP, LEV, TET, NIT, SXT, MEM, IMI |
| 10 | Dehbashi, S. 2020 | 12/2017 to 9/2018 | Iran | ND | ND | 35 M, 53 F | 88 | KB, DDST | CAZ, DOR, MEM, IMI, CPM, CIP, GEN, AMK, NOR, AZT, TZP, TOB, PIP |
| 11 | El-Mahdy, R. 2019 | 9/2018 to 4/2019 | Egypt | ND | ND | ND | 80 | KB, Broth microdilution, DDST, PCR | POL, COL, CAZ, CPM, PIP, TZP, AZT, TOB, GEN, AMK, LEV, CIP |
| 12 | Falodun, O. I. 2021 | 4/2018 to 5/2018 | Nigeria | 147 | ND | ND | 82 | KB, DDST, PCR | AMC, CAZ, CPM, AZT, IMI, GEN, CIP, TMP |
| 13 | Jarjees, K. K. 2021 | 5/2019 to 11/2019 | Iraq | 650 | ND | ND | 62 | VITEK 2, PCR | TIC, TCC, PIP, TZP, CAZ, CPM, IMI, MEM, AMK, GEN, TOB, CIP |
| 14 | Jayakumar, N. 2020 | 2/2018 to 1/2019 | India | ND | All group | 69 M, 65 F | 134 | KB, DDST | GEN, AMK, CIP, LEV, CAZ, CPM, AMP, PIP, TZP, AZT, IMI, MEM |
| 15 | Kaluba, C. K. 2021 | 3/2018 to 6/2019 | Zambia | 384 | ND | ND | 84 | KB | CPM, CAZ, CIP, GEN, IMI, TZP, AMK |
| 16 | Karampoor, M. 2022 | 2018 to 2019 | Iran | ND | ND | 75 M, 42 F | 117 | KB, DDST, PCR | GEN, AMK, TOB, CAZ, PIP, IMI, CIP |
| 17 | Khademi, F. 2021 | 6/2019 to 4/2021 | Iran | ND | ND | ND | 103 | KB, PCR | PIP, TZP, TCC, CAZ, CPM, AZT, DOR, IMI, MEM, GEN, TOB, AMK, CIP, NOR, LEV, OFX, NET, LOM |
| 18 | Kumari, N. 2022 | 3/2019 to 12/2019 | India | 101 | All group | ND | 11 | KB, DDST, PCR | CAZ, TZP, AZT, OFX, GEN, AMK, TOB, MEM, IMI |
| 19 | Lee, Y. L. 2019 | 2018 | Taiwan | 1184 | ND | ND | 252 | VITEK 2, PCR  KB, PCR | CAZ, CPM, TZP, IMI, MEM, CIP, LEV, GEN, AMK, COL |
| 20 | Liu, M. 2020 | 9/2018 to 10/2018 | China | ND | ND | ND | 113 | VITEK 2, PCR  KB, PCR | AMK, AZT, CAZ, CIP, CPM, GEN, IMI, LEV, MEM, PIP, TOB, TZP |
| 21 | Manyahi, J. 2020 | 4/2018 to 5/2018 | Tanzania | 402 | All group | 235 M, 167 F | 11 | KB, DDST | GEN, CTR, CAZ, CTX, AMC, AMK, MEM, TZP, CIP, SXT |
| 22 | Martinez-Miranda, R. 2020 | 8/2018 to 1/2019 | Mexico | ND | ND | ND | 19 | VITEK 2, KB | TCC, TZP, CAZ, CPM, IMI, MEM, LEV, CIP, AMK, COL |
| 23 | Mohamed, A. H. 2022 | 1/2019 to 12/2021 | Somalia | 99 | All group | 62 M, 35 F | 19 | KB, DDST | CFZ, CTX, FOX, CFX, CAZ, CPM, CFM, AMP, AMC, GEN, AMK, SXT, CIP, LEV, IMI, MEM, ETP, TZP, TGC, COL |
| 24 | Morroni, G. 2022 | 10/2018 to 3/2019 | Italy | ND | ND | ND | 317 | VITEK 2, Broth microdilution, PCR | CXT, TZP, MEM, CPM, AMK |
| 25 | Muhammad, A. 2020 | 2/2019 to 9/2019 | Pakistan | ND | All group | 96 M, 66 F | 162 | KB, DDST | CIP, GEN, IMI, MEM, CAZ, TZP, AMK |
| 26 | Mustafai, M. M. 2023 | 2/2022 to 4/2022 | Pakistan | 384 | All group | 210 M, 174 F | 10 | KB, DDST, PCR | AMK, GEN, CAZ, CPM, MEM, IMI, TZP |
| 27 | Namaei, M. H. 2021 | 1/2018 to 5/2019 | Iran | 122 | All group | 70 M, 52 F | 66 | KB, Broth microdilution, DDST, PCR | TZP, AMK, AZT, LEV, GEN, MIN, CAZ, CTX, CPM, IMI, MEM, COL |
| 28 | Nasser, M. 2020 | 7/2018 to 12/2018 | Yemen | 200 | All group | 77 M, 21 F | 98 | VITEK 2, KB, DDST, PCR | GEN, TOB, AMK, CAZ, CPM, AZT, TZP, IMI, MEM, CIP |
| 29 | Ngoi, S. T. 2021 | 10/2019 to 7/2020 | Malaysia | 378 | All group | 30 M, 24 F | 54 | VITEK 2, KB, Broth microdilution, PCR | AMK, CIP, CTX, CAZ, IMI, MEM |
| 30 | Pandey, R. 2021 | 2/2018 to 7/2018 | Nepal | 452 | All group | 203 M, 249 F | 84 | KB, DDST, PCR | AMK, CIP, CAZ, CPM, TZP, MEM, COL, POL |
| 31 | Park, Y. 2022 | 3/2020 to 2/2021 | South Korea | ND | ND | ND | 124 | Broth microdilution, PCR | IMI, MEM, LEV, CIP, GEN, CAZ, TZP, AZT |
| 32 | Rad, Z. R. 2021 | 2019 to 2020 | Iran | ND | All group | 36 M, 34 F | 70 | KB, Broth microdilution, DDST, PCR | MEM, IMI, DOR, TOB, CAZ, AZT, TZP, GEN, AMK, CIP, COL |
| 33 | Ruekit, S. 2022 | 2017 to 2018 | Thailand | 431 | ND | ND | 20 | VITEK 2, PCR | ETP, IMI, MEM, CFZ, CEP, CFX, FOX, CTX, CAZ, CTR, CPM, AZT, AMP, SAM, AMCC, PIP, TZP, CIP, LEV, NOR, TMP, SXT, GEN, AMK, TOB, TET, NIT, CHL, COL |
| 34 | Saleem, S. 2020 | 2017 to 2018 | Pakistan | 108 | ND | ND | 88 | KB, DDST, PCR | CIP, CAZ, AZT, IMI, TGC, AMK, COL |
| 35 | Shalmashi, H. 2022 | 2018 to 2020 | Iran | ND | ND | 39 M, 61 F | 100 | KB, DDST, PCR | CAZ, CPM, IMI, GEN, AZT, CIP, AMK, TZP |
| 36 | Shukla, S. D. 2021 | 1/2019 to 2/2021 | India | 502 | >10 years old | ND | 115 | KB, DDST | COL, AMK, TZP, PIP, GEN, MEM, IMI, CIP, TCC, AZT, CPM, CAZ, POL |
| 37 | Tilahun, M. 2022 | 2/2021 to 8/2021 | Ethiopia | 423 | All group | ND | 46 | KB, DDST | AMK, TZP, CIP, CAZ, GEN, MEM, AZT |
| 38 | Tran, H. D. 2022 | 4/2018 to 5/2019 | Vietnam | 254 | >15 years old | 124 M, 130 F | 21 | VITEK 2 | AMP, AMC, PIP, TZP, SAM, FOX, CFX, CTX, CTR, CAZ, CPM, CIP, LEV, GEN, AMK, IMI, MEM, SXT, TET, DOX, TGC, COL |
| 39 | Yong, Y. K. 2021 | 3/2019 to 6/2019 | Malaysia | 127 | All group | 88 M, 39 F | 33 | KB, PCR | AMK, TOB, TZP, IMI, MEM, CFZ, CAZ, CPM, LEV, CIP |
| 40 | Zahoor, Z. 2023 | 4/2021 | Pakistan | 150 | Neonates | ND | 12 | KB, PCR | AMC, GEN, CIP, CFM, MEM, FOS, TGC, AMK, SXT, CAZ |

ND, no data; KB, Kirby-Bauer disk diffusion; DDST, double disk synergy test; PCR, polymerase chain reaction; AMC, amoxicillin and clavulanic acid; AMK, amikacin; AMP, ampicillin; AMX, amoxicillin; AZT, aztreonam; CAZ, ceftazidime; CAR, carbenicillin; CEP, cephalothin; CFM, cefixime; CFR, cefadroxil; CFX, cefuroxime; CFZ, cefazolin; CHL, chloramphenicol; CIP, ciprofloxacin; COL, colistin/polymyxin E; CPD, cefpodoxime; CPM, cefepime; CTR, ceftriaxone; CTX, cefotaxime; CXT, ceftolozane and tazobactam; CZA, ceftazidime and tazobactam; DOR, doripenem; DOX, doxycycline; ETP, ertapenem; ERY, erythromycin; FOS, fosfomycin; FOX, cefoxitin; FUS, fusidic acid; GEN, gentamicin; IMI, imipenem; LEV, levofloxacin; MEM, meropenem; MIN, minocycline; NAL, nalidixic acid; NIT, nitrofurantoin; NOR, norfloxacin; OFX, ofloxacin; OXA, oxacillin; PIP, piperacillin; POL, polymyxin B; SAM, ampicillin and sulbactam; SXT, sulfamethoxazole and trimethoprim; TCC, ticarcillin and clavulanic acid; TET, tetracycline; TGC, tigecycline; TIC, ticarcillin; TMP, trimethoprim; TOB, tobramycin; TZP, piperacillin and tazobactam
