# Supplementary material for: Current trends in the epidemiology of multidrug-resistant and beta-lactamase-producing Pseudomonas aeruginosa in Asia and Africa: a systematic review and meta-analysis
Source: PeerJ. 2025 Feb 24;13:e18986. doi: 10.7717/peerj.18986 (PMC11867037; doi:10.7717/peerj.18986)
Supplement: Supplemental Information 4 [file peerj-13-18986-s004.docx]

**Current trends in the epidemiology of multidrug-resistant and beta-lactamase-producing *Pseudomonas aeruginosa* in Asia and Africa: a systematic review and meta-analysis**

1. The rationale for conducting the systematic review / meta-analysis:

The increasing prevalence of antibiotic-resistant *P. aeruginosa* poses a significant threat to public health, necessitating the implementation of robust surveillance programs and antibiotic stewardship initiatives. These efforts are critical for monitoring resistance trends, identifying outbreaks, and ensuring the rational use of antibiotics to preserve the efficacy of existing antimicrobial agents. To support these initiatives, this systematic review and meta-analysis aims to provide a comprehensive and up-to-date pooled prevalence estimate of antibiotic-resistant *P. aeruginosa* in Asia and Africa, synthesizing data published in the last five years.

1. The contribution that it makes to knowledge in light of previously published related reports, including other meta-analyses and systematic reviews:

This systematic review and meta-analysis make a significant contribution to the existing body of knowledge by providing an updated and comprehensive pooled prevalence estimate of antibiotic-resistant *P. aeruginosa*, based on data published in the last five years. Unlike previous meta-analyses, which have focused on limited geographic regions or earlier time frames, this review synthesizes recent data, offering a more current and accurate understanding of resistance trends. For example, our analysis revealed that the estimated prevalence of pseudomonal infections across various age groups from different settings in Asia and Africa was 22.9% (95% CI: 14.4–31.4), with significant regional variation compared to a report publish in 2018 by Restrepo et al., which reported at 11.3% from the community-acquired pseudomonal infections. The majority of studies included in this SRMA were conducted in Pakistan, where the pooled prevalence of *P. aeruginosa* in Pakistan was calculated at 21.3%, notably higher than the prevalence estimate in a study from Pakistan in 2019, which reported an estimate of 9.3% (Farooq et al. 2019). Furthermore, our meta-analysis revealed that the current prevalence of MDR *P. aeruginosa* was estimated at 46.0%, slightly higher than previously reported at 38.3% of MDR *P. aeruginosa* bloodstream infections in 2020 (Recio et al. 2020) and substantially higher than the report from a 2019–2020 single-center retrospective case control study in the USA recorded at 29.8% (Yang et al. 2023). As for XDR, our analysis revealed that the current pooled prevalence of XDR *P. aeruginosa* was markedly high, with an estimation at 19.6%, compared to the majority of European countries reported resistance rates of around 10% (P. et al. 2019). In addition, the pooled prevalence of ESBL-producing *P. aeruginosa* was 33.4%, while the prevalence of MBL-producing strains was found to be 16.0% and to our knowledge, our SRMA is the first to address the pooled prevalence of ESBL- and MBL-producing strains of *P. aeruginosa* from the past five years in Asia and Africa. By addressing gaps—such as the inclusion of ESBL- and MBL-producing strains—this work not only enhances the epidemiological understanding of *P. aeruginosa* but also informs antibiotic stewardship initiatives and surveillance programs. Consequently, the findings have the potential to shape future clinical practices and policies aimed at controlling the spread of antibiotic resistance.
